# Machine-Learning-Based Probabilistic Model and Design-Oriented Formula of Shear Strength Capacity of UHPC Beams

**DOI:** 10.3390/ma18204800

**Published:** 2025-10-21

**Authors:** Kun Yang, Jiaqi Xu, Xiangyong Ni

**Affiliations:** 1 CNEC Innovation Technology Co., Ltd., Shanghai 201702, China; yangkun@cnecc.com; 2 Shanghai Jianke Prestressed Construction Engineering Co., Ltd., Shanghai 200030, China; 3 Department of Civil Engineering, Shanghai University, Shanghai 200444, China

**Keywords:** machine learning, UHPC beam, shear strength capacity (SSC), hyperparameter optimization, weight, 95% confidence interval

## Abstract

Designing UHPC beams for shear is challenging because many factors—geometry, concrete strength, fibers, and stirrups—act together. In this study, we compile a large, curated database of laboratory tests and develop machine learning models to predict shear capacity. The best models provide accurate point predictions and, importantly, a 95% prediction band that tells how much uncertainty to expect; in tests, about 95% of results fall inside this band. For day-to-day design, we also offer a short, design-oriented formula with explicit coefficients and variables that can be used in a spreadsheet. Together, these tools let engineers screen options quickly, check designs with an uncertainty margin, and choose a conservative value when needed. The approach is transparent, easy to implement, and aligned with common code variables, so it can support preliminary sizing, verification, and assessment of UHPC members.

## 1. Introduction

Ultra-high-performance concrete (UHPC) is a next-generation cementitious composite, and it is renowned for its exceptional mechanical properties and long-term durability. With compressive strengths exceeding 150 MPa, superior ductility, low permeability, and enhanced crack resistance, UHPC has attracted growing interest for its potential in structural engineering applications [1]. In the field of civil engineering, its significance lies in its capacity to enhance structural durability and safety, reduce material consumption, and align with sustainable development goals [2,3]. As fundamental load-bearing components, UHPC beams are widely utilized in bridges, residential buildings, commercial facilities, and other infrastructure. A thorough understanding of their fundamental mechanical behavior is essential for advancing UHPC’s practical application in engineering contexts [4]. Recent developments in resilient and self-centering structural systems [5] further demonstrate the growing demand for high-performance materials, such as UHPC, in improving post-earthquake recoverability and energy-dissipation capacity. While the flexural strength of UHPC beams can be reliably estimated using the plane section assumption, accurately predicting their shear strength capacity (SSC) remains challenging. This difficulty arises primarily from the incorporation of fibers, which significantly alter the shear behavior by bridging cracks and enhancing post-cracking resistance. The fiber bridging effect at inclined shear cracks plays a pivotal role and cannot be neglected. Consequently, traditional shear design models often fall short in accurately estimating the SSC of UHPC beams. Furthermore, the SSC is influenced by a complex interplay of multiple parameters, including cross-sectional geometry, shear span ratio, material strength, reinforcement ratio, and fiber characteristics. These factors are strongly interrelated, making it difficult for conventional analytical models to capture the underlying nonlinear relationships effectively. In response to these challenges, this study leverages big data and employs machine learning (ML) algorithms to develop a predictive model for the SSC of UHPC beams. The model is further extended into a probabilistic framework by constructing confidence intervals based on the predictive performance of each ML approach. Finally, a simplified empirical formula for estimating the SSC is proposed, derived from a comprehensive experimental database.

Numerous studies have been conducted to investigate the shear strength capacity (SSC) of UHPC beams, including the development of empirical formulas based on experimental data, adaptations of plastic theory, and modifications to the pressure field theory. Voo et al. [6,7] performed shear tests on I-shaped UHPC beams and proposed a simplified SSC model grounded in plastic theory, specifically the Two Bounds Theory. This method assumes a uniform fiber distribution and incorporates fiber reinforcement parameters to account for fiber-induced shear enhancement. The validity of the proposed model was confirmed through experimental results. Baby et al. [8,9,10] tested 11 I-shaped UHPC beam specimens, with variables including UHPC type, the presence or absence of stirrups, and the type of longitudinal reinforcement (prestressed tendons vs. conventional steel bars). To address the lack of reliable tensile performance data for UHPC, a variable participation model was employed to estimate post-cracking tensile strength based on fiber–matrix interaction. Subsequently, a modified pressure field theory was used to develop an SSC prediction method tailored to UHPC beams. Xu et al. [11] conducted shear tests on nine T-shaped UHPC beams and reported that using existing design codes to estimate SSC often results in overly conservative predictions. A new SSC formula was proposed based on regression analysis of the experimental data. Zheng et al. [12] performed shear tests on nine prestressed thin-walled UHPC box beams and derived an SSC model by decomposing shear resistance into three components: fiber concrete, stirrups, and prestressing force. Qi et al. [13,14] investigated 11 T-shaped UHPC beams, formulating a theoretical model that considers the combined shear contribution from concrete, stirrups, and fibers. The model’s predictions showed good agreement with experimental results. Qiu et al. [15] compiled a shear test database for UHPC beams and evaluated the predictive accuracy of SSC formulas in various national design codes. The findings revealed that most codes tend to be conservative to varying degrees. Despite these advances, most of the aforementioned models were developed and validated using relatively small experimental datasets. The highest reported prediction accuracy was R^2^ = 0.80, as noted by Qiu et al. [15], indicating the need for further enhancement through data-driven and probabilistic approaches.

In recent years, machine learning (ML) has shown strong potential for predicting the shear strength capacity (SSC) of reinforced-concrete (RC) components by capturing nonlinear, coupled effects that are difficult to express analytically [16]. Beyond correlation measures such as R^2^, recent studies on RC shear walls and columns increasingly report error magnitudes and dispersion/bias metrics (e.g., MAE, RMSE, MAPE, prediction-to-test ratios, CoV) to support engineering reliability [17,18,19,20,21,22,23,24,25]. For shear walls, data-driven and stacked learners demonstrate that complementing R^2^ with absolute/relative error gives a fuller view of robustness under varying geometry, concrete strength, reinforcement ratios, and loading conditions [17,18,19,20]. For columns, joints, slab–column punching, and RC beams, similar practice—benchmarking ML against empirical formulas using MAE/RMSE/MAPE or ratio/CoV—has been adopted to quantify practical accuracy and bias [21,22,23,24,25].

Focusing on UHPC beams, Ye et al. (2023) [26] trained ten algorithms on 532 tests and used SHAP to interpret feature effects (geometry and shear span ratio dominant); CatBoost reached R^2^ = 0.943, and the study benchmarks models with error metrics in addition to R^2^. Ergen and Katlav (2024) developed interpretable DL pipelines (LSTM/GRU with optimizers) and reported RMSE/MAE/MAPE together with R^2^ for model comparison [27]. Earlier, Solhmirzaei et al. (2020) proposed a unified ML framework for UHPC-beam failure-mode classification and SSC prediction from 360 tests; their genetic-programming expression achieved R^2^ ≈ 0.92 with deployment-oriented reporting beyond correlation only [28]. Ni and Duan (2022) [29] curated 200 specimens and compared ANN/SVR/XGBoost; besides R^2^ (0.8825/0.9016/0.8839), they explicitly reported prediction-to-test means (1.08/1.02/1.10) and coefficients of variation (0.28/0.21/0.28), directly quantifying bias and dispersion [29].

Broader RC wall studies reinforce this error-aware reporting. Tian et al. [19] showed that a stacking model achieved R^2^ = 0.98 with CoV = 0.147 on the test set, while a DNN baseline reached RMSE ≈ 263 kN and R^2^ ≈ 0.95; independent ML models had R^2^ = 0.88–0.95 and CoV = 0.179–0.651 [16]. Zhang et al. (2022) [18] reported failure-mode accuracy > 95%, a mean predicted-to-tested strength ratio ≈ 1.01, and a predicted-to-tested ultimate drift ratio ≈ 1.08, thereby explicitly addressing bias and dispersion in addition to strength prediction [18]. Across other RC components—columns [21], beam–column joints [22], slab–column punching [23], conventional RC beams [24], and synthetic-fiber RC beams [25]—recent works likewise present MAE/RMSE (and, where available, MAPE or ratio/CoV) alongside R^2^, supporting a more engineering-relevant assessment of robustness and practical reliability.

Recent contributions on UHPC + ML advance three complementary fronts. First, for the shear capacity of beams and joints, multiple studies show that interpretable ensembles and gradient-boosting methods (e.g., CatBoost/XGBoost/LightGBM) provide state-of-the-art accuracy and transparent feature attributions, often outperforming plain MLPs on tabular data [30,31,32,33,34]; related work on the punching shear of post-tensioned UHPC slabs extends these benefits to slab systems [32]. Second, for compressive and flexural strengths, recent papers corroborate the advantage of boosting pipelines and introduce CNN/NN variants and SHAP-based explanations for practical insight [35,36,37,38]. Third, explainable modeling and interface behavior (e.g., steel–UHPC slip) further emphasize engineering interpretability [38,39]. Across these studies, common themes include model transparency (via SHAP) and competitive performance of boosting, while typical gaps are heterogeneous or non-grouped validation protocols, limited reporting of calibrated uncertainty, and incomplete statements of applicability domains.

Although the above studies have shown that ML models can provide reliable predictions for SSC in both RC and UHPC members, several limitations remain, as follows:

(1) Underutilization of model complementarity:

Most existing studies focus on comparing individual ML models rather than integrating their complementary strengths. Future work should consider ensemble strategies that combine multiple ML algorithms to enhance both accuracy and robustness.

(2) Lack of uncertainty modeling:

The inherent uncertainty in SSC prediction has not been fully addressed. Probabilistic modeling techniques—such as prediction-interval construction (e.g., residual- or quantile-based) and supervised machine learning regression with cross-validated calibration—should be incorporated to better quantify prediction reliability.

(3) Incomplete evaluation across model types:

There is insufficient comparative analysis between different categories of ML models (e.g., single learners, ensemble methods, and deep learning). A more systematic evaluation is needed to identify the most suitable models for SSC prediction in UHPC beams.

(4) Limited accuracy of current design codes:

Existing national and international design specifications often fail to provide precise SSC predictions for UHPC beams, indicating the need for more data-driven, refined formulations.

To address these limitations, this study first establishes an experimental database containing 563 UHPC beam specimens exhibiting shear failure. Three categories of ML models—single, ensemble, and deep learning—are developed and evaluated under both default and optimized hyperparameter configurations. Model performances are then compared, and their outputs are integrated using a weighted ensemble approach based on prediction accuracy. A 95% confidence interval is constructed to capture uncertainty in SSC predictions. Finally, leveraging both the established database and existing Chinese code formulations, a simplified empirical equation is proposed for engineering applications.

Novelty and Contributions: This work advances UHPC beam shear capacity prediction in four concrete ways: (i) we assemble a large-scale database of 563 shear-failing UHPC beams spanning wide geometric, reinforcement, fiber, and loading attributes; (ii) we propose a multi-metric performance-weighted stacking model that fuses GBR, XGBoost, LightGBM, and CatBoost using MSE, MAE, RMSE, and R^2^ as weighting criteria (Equations (1)–(3)), achieving R^2^ = 0.96 with CoV = 0.17 on the test set; (iii) we develop a residual-based probabilistic framework that yields 95% confidence intervals with 95.1% empirical coverage; and (iv) we derive a design-oriented empirical formula calibrated on the database that outperforms multiple code provisions with R^2^ = 0.83. Collectively, the pipeline—from feature selection and cross-family model benchmarking to stacking, uncertainty quantification, and a practical design equation—provides a unified, data-driven, and reliability-aware solution for UHPC beam shear design.

## 2. Framework of Probabilistic Models

**Figure 1:**
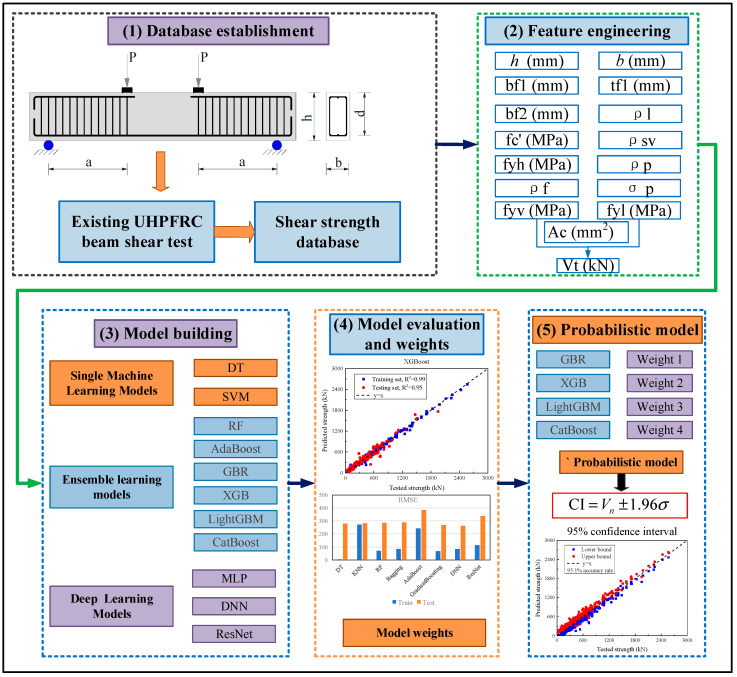
Framework of probabilistic models.

Figure 1 outlines the methodological framework for developing a probabilistic prediction model for the SSC of UHPC beams. The proposed framework consists of five key steps:

Step 1: Experimental Database Compilation

A comprehensive experimental database comprising 563 UHPC beam specimens exhibiting shear failure was constructed by aggregating data from the published literature.

Step 2: Feature Engineering

To identify the most influential input variables, feature importance scores were derived using a random forest (RF) regression algorithm. These scores were further analyzed in conjunction with Pearson’s correlation coefficients to eliminate redundant or weakly correlated features, thereby enhancing model efficiency through dimensionality reduction.

Step 3: Model Development

Three categories of ML models—single, ensemble, and deep learning—were trained to predict the SSC of UHPC beams. An initial assessment was carried out using default settings for all models, and subsequent performance improvements were achieved through hyperparameter optimization.

Step 4: Model Performance Assessment and Weight Assignment

Model accuracy was assessed using standard performance metrics (e.g., R^2^, MAE, RMSE). The four best-performing models were selected based on test-set performance, and relative weights were assigned proportionally to their predictive capabilities.

Step 5: Probabilistic Model Construction

A weighted ensemble approach was used to integrate the selected models. By analyzing the residuals of each specimen’s predicted and actual SSC values, a 95% confidence interval was established to quantify prediction uncertainty. Using the curated database and drawing upon design provisions in the Chinese code, a simplified empirical formula was developed to estimate SSC for practical engineering use.

This framework ensures not only accurate point predictions of SSC but also probabilistic bounds that reflect real-world variability, making it suitable for both academic and design applications.

## 3. Experimental Database

### 3.1. Shear Test of UHPC Beams

**Figure 2:**
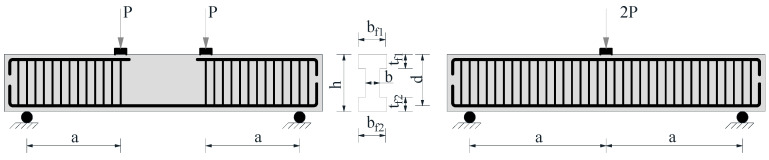
Shear tests of UHPC beams.

This study utilizes an experimental database containing a total of 563 UHPC beam specimens that failed in shear, and the UHPC beam shear test setup is shown as Figure 2. Among the compiled studies, 523 specimens were taken from the database of Ye et al. (2023) [26], which aggregates 68 sources including Yang et al. [4], Voo et al. [6], Voo et al. [7], Baby et al. [9], Zheng et al. [12], Qi et al. [14], Xu et al. [36], Bunje et al. [40], Tuchlinski et al. [41], Graybeal [42], Hegger et al. [43], Wu et al. [44], Telleen et al. [45], Magureanu et al. [46], Diao et al. [47], Sarsam et al. [48], Yang et al. [49], Li et al. [50], Lee et al. [51], Aziz et al. [52], Chen [53], Al-Hassani et al. [54], Kamal et al. [55], Hussein et al. [56], Jin et al. [57], Deng et al. [58], Bertram [59], Thiemicke [60], Luo et al. [61], Lim et al. [62], Zagon et al. [63], Lee [64], Pansuk et al. [65], Mészöly et al. [66], Pourbaba et al. [67], Yousef et al. [68], Mohammed et al. [69], Ji et al. [70], Liang et al. [71], Chen et al. [72], Jin et al. [73], Cao et al. [74], Zheng et al. [75], Ahmed et al. [76], Jin et al. [77], Schramm et al. [78], Hasgul et al. [79], Yavaş et al. [80], Tong [81], Ma et al. [82], Yang [83], Zhang [84], Yavaş et al. [85], Ridha et al. [86], Ţibea et al. [87], Bermudez et al. [88], Bae et al. [89], Metje et al. [90], Wen [91], Lu et al. [92], Luo et al. [93], El-Helou et al. [94], Chen et al. [95], Ma et al. [96], Li et al. [97], Cao et al. [98], Feng et al. [99], and Ye et al. [100]. The per-specimen details of these 523 UHPC beams are summarized in Ye et al. [26] and are not re-tabulated here to avoid duplication. In addition, we curated new specimens from recent studies [101,102,103,104,105]; a summary is given in Table 1, and per-specimen details are provided in Table 1.

All specimens were subjected to shear testing under various loading conditions and reinforcement configurations, ensuring a broad representation of UHPC structural behavior. In both three-point bending and four-point bending, the ultimate shear capacity is extracted as the support reaction at failure. Under the symmetric setups shown in Figure 2, this reaction is the same in the two configurations; therefore, the shear capacity obtained from either scheme is equivalent. The different bending-moment distributions are accounted for in our analysis through the shear span ratio considered elsewhere, so the choice of loading scheme does not, by itself, affect the reported shear capacity.

### 3.2. Experimental Database Description

Each specimen in the database is characterized by a set of geometric, material, and reinforcement parameters. Key geometric attributes include beam height (*h*, unit: mm), width (*b*, unit: mm), the area of UHPC beam cross-section (*A*_c_, unit: mm^2^), flange widths (*b_f_*_1_, *b_f_*_2_, unit: mm), and thicknesses (*t_f_*_1_, *t_f_*_2_, unit: mm). Reinforcement-related parameters encompass the longitudinal reinforcement ratio (*ρ_l_*), yield strength of longitudinal reinforcement (*f_sy_*, unit: MPa), prestressing ratio (*ρ_p_*), and prestressing stress (*σ_p_*, unit: MPa). Shear reinforcement characteristics such as stirrup spacing (s), stirrup ratio *(ρ_sv_*), and yield strength (*f_sv_*, unit: MPa) are also included. Material properties are represented by UHPC compressive strength (*f_c_*, unit: MPa), fiber volume fraction (*ρ_f_*), fiber length (*l_f_*), and fiber diameter (*d_f_*). The shear span ratio (*m*) and the experimentally obtained shear strength capacity (*V_u_*, unit: kN) are included as the target output variable.

**Figure 3:**
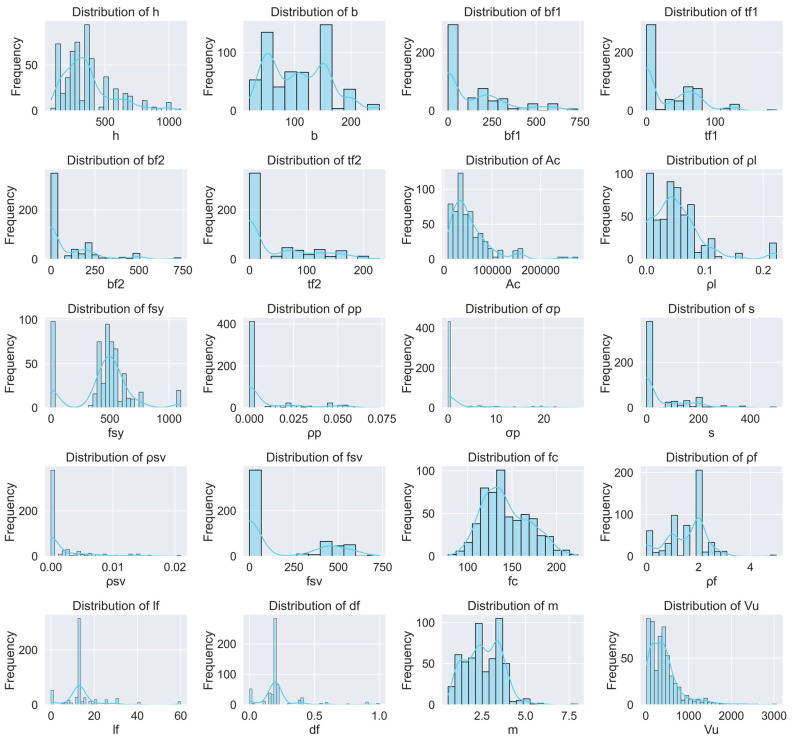
Distribution diagram of each parameter.

Table 2 presents a statistical summary of all input and output variables, and the distribution of each parameter is shown in Figure 3. The average beam height (*h*) is 361 mm, with a standard deviation of 194 mm, indicating a wide range of specimen sizes. The mean value of *V_u_* is 433.1 kN, spanning from as low as 18.2 kN to a maximum of 3053.0 kN, demonstrating significant variability in shear resistance. Similarly, the fiber content, ρf, ranges from 0 to 5%, and stirrup spacing varies from 0 to 500 mm, reflecting diverse design scenarios across the dataset. Descriptive statistics such as minimum, maximum, mean, standard deviation, and interquartile ranges (25%, 50%, 75% quantiles) were calculated for all features to understand their distribution. For example, the 75th percentile of *h* is 400 mm, indicating that most specimens are medium-to-large in height. In contrast, stirrup spacing is mostly concentrated in the lower quartile, implying that closely spaced stirrups were common in the dataset.

We first harmonized all variables to consistent units (dimensions in mm, strengths in MPa, loads in kN). In particular, stirrup spacing (*s*) is reported in mm, and fiber content (ρf) is expressed as volume fraction (%). No ad hoc filtering was applied specifically on (*s*) or (ρf); the database preserves their full reported ranges after routine data quality checks. For modeling, tree-based learners (GBR, XGBoost, LightGBM, CatBoost) used the unscaled (unit-harmonized) predictors. Neural network models employed z-score standardization for all continuous predictors (including (*s*) and (*ρ*_f_)), computed only on the training set and then applied to validation/test data to prevent information leakage: *x*^std^ = (*x* − *u*_train_)/*σ*_train_. Transverse reinforcement was encoded by (s) and, where available, the volumetric stirrup ratio (*ρ*_sv_), ensuring that both spacing and volumetric content are represented.

## 4. Feature Engineering and Model Building

### 4.1. Characteristic Parameters

**Figure 4:**
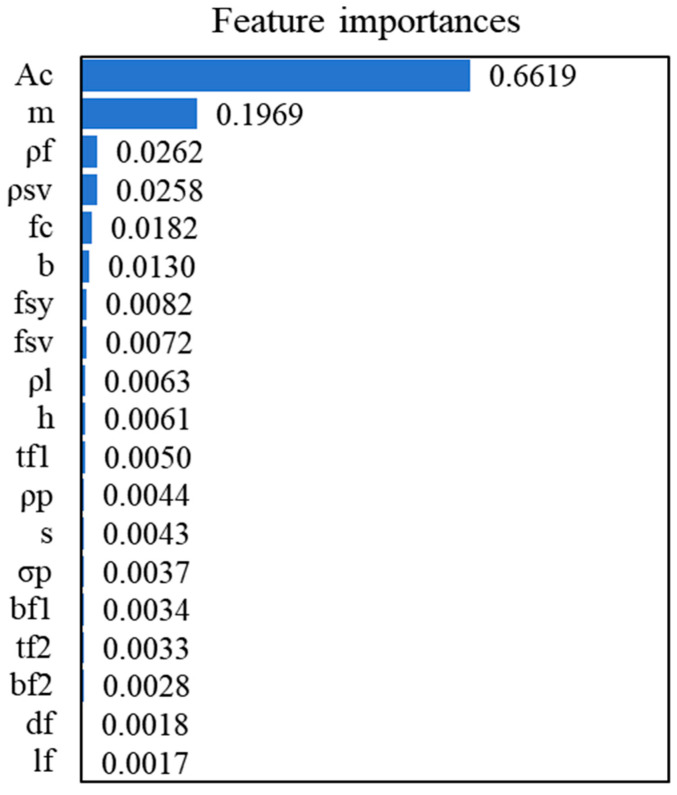
Importance coefficient of each characteristic parameter.

The importance of features is evaluated using a random forest regression model to obtain the features that affect significantly the prediction of the target variable from the original feature set. The UHPC beam dataset was partitioned into training and testing subsets via the train_test_split function, after which a random forest model was instantiated and trained using RandomForestRegressor. After the model training is completed, the importance score of each feature is calculated using the ‘feature_importances_’ attribute. These values indicate the relative influence of each input variable on the target output. Finally, these important features are extracted, as shown in Figure 4. Feature importance is the impurity-based importance from a RandomForestRegressor, averaged across grouped-CV folds and normalized to 1; a permutation-importance check on held-out folds yielded a consistent ranking.

*A_c_* is as high as 0.66187, highlighting its significant influence on model performance. Secondly, the shear span ratio m also has a significant effect with a significance of 0.196879. The importance of other features such as *ρ_f_* (0.026242), *ρ_sv_* (0.025779), and *f_c_* (0.01818) gradually weakened, indicating that their contribution to the model prediction was limited. Overall, when building subsequent machine learning models, priority was given to high-importance features such as *A_c_* and *m* to improve the performance and safety of the final product.

**Figure 5:**
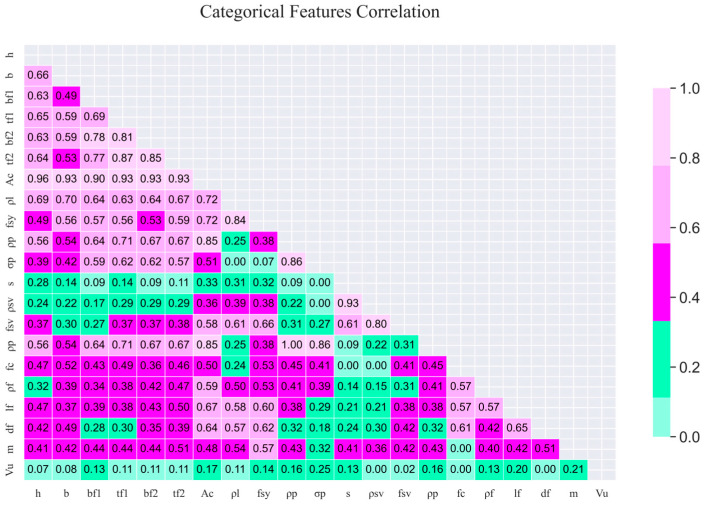
Correlation coefficients between parameters.

Figure 5 shows the correlation matrix between multiple parameters and their respective correlation coefficients. The analysis results show that *A_c_* has significant correlations with several key parameters. A key observation is that the feature importance results also show that *A_c_* is the most important feature affecting the model output, with an importance score as high as 0.66187, which complements its high correlation and shows the dominant influence of *A_c_* on the SSC of UHPC beams. Meanwhile, although *b_f1_* has a high correlation with other features (such as *h* and *t_f_*_1_) (0.59 and 0.83, respectively), its importance score is 0.007195, indicating that it may be relatively minor in enhancing the SSC of UHPC beams.

Based on the above results, the features with low importance (*d_f_* and *l_f_*, with importances of 0.00179 and 0.001704, respectively) were eliminated in the subsequent analysis to simplify the model and reduce the computational complexity.

### 4.2. ML Models

The SSC prediction models of UHPC beams were constructed using single models, ensemble models, and deep learning models. Single models, such as decision trees (DT), are widely used because of their simple structure and easy interpretation. Single models provide a quick baseline in the preliminary analysis and model validation stages, and they can intuitively show the relationship between features and target variables. However, single models usually have limited performance in dealing with complex nonlinear relationships and high-dimensional data. As a result, the prediction results obtained by single models often fail to achieve optimal performance. Ensemble models, such as RF, extreme gradient boosting (XGBoost), LightGBM, and CatBoost, can effectively improve the accuracy and stability of the model by combining the prediction results of multiple base learners. Ensemble methods avoid overfitting by reducing variance and improve the ability to capture complex data patterns. For example, XGBoost and LightGBM not only provide high prediction performance but also can handle real-time performance of large-scale data sets. This innovativeness makes ensemble models an important tool for dealing with complex interactions between features and improving the accuracy of shear strength capacity prediction. Deep learning models such as multi-layer perceptron (MLP), deep neural network (DNN), and residual network (ResNet) have shown significant advantages in processing highly complex nonlinear relationships. They automatically extract features through multi-layer structures and can capture subtle patterns in data. In addition, the ResNet model using residual connections can train deeper networks, solving the gradient vanishing problem in deep network training and further improving performance.

**Figure 6:**
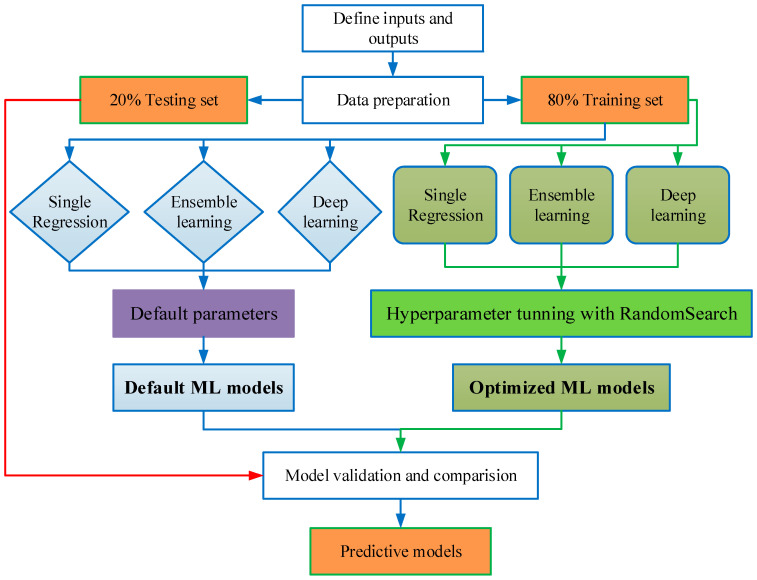
Training process of each machine learning model.

Combining the characteristics of the models mentioned above, in the calculation of the SSC of UHPC beams, the use of a single model can quickly establish a baseline, while the ensemble model improves accuracy and stability and adapts to the needs of complex features. The deep learning model provides a powerful ability to process high-dimensional and complex patterns. Therefore, when constructing this calculation model, by gradually adopting the single, ensemble, and deep learning models, as shown in Figure 6, their strengths can be effectively integrated to enhance model accuracy, resulting in a more robust prediction scheme for UHPC beam shear strength.

All models were implemented using Python with the scikit-learn, lightgbm, catboost, xgboost, and tensorflow.keras libraries. The training–testing split was conducted using an 80:20 ratio. Initial training was performed with default hyperparameters to establish baselines before optimization. The rationale behind this tiered modeling strategy is to evaluate the trade-offs between simplicity, interpretability, and predictive power, ultimately enabling the integration of complementary strengths across model types.

## 5. Model Evaluation

### 5.1. ML Models with Default Parameter Input

#### 5.1.1. Single ML Models with Default Parameter Input

**Figure 7:**
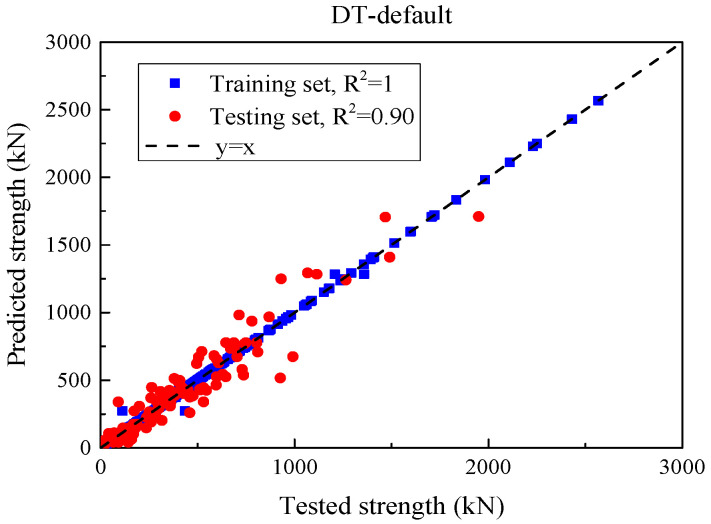
Comparison of DT model prediction values and experimental values for each beam specimen under default parameter input.

Figure 7 compares the experimental SSC with DT predictions under default hyper-parameters, and Table 3 summarizes the accuracy on the training and test partitions. On the training set, DT attains MSE = 148.3 kN^2^, MAE = 1.5 kN, RMSE = 12.2 kN, and R^2^ = 1.00, indicating an almost perfect in-sample fit. However, on the test set, MSE increases to 11,829.6 kN^2^, MAE to 76.0 kN, and RMSE to 108.8 kN, while R^2^ decreases to 0.90. Interpreting these metrics, MAE (76 kN) reflects the typical absolute error per specimen, RMSE (108.8 kN) highlights the presence of occasional larger errors (since RMSE weights large deviations more), and MSE quantifies the same in squared units. Thus, despite a high test R^2^, the absolute errors are non-negligible, and the large train–test gap evidences overfitting of a high-variance tree that memorizes training patterns but does not generalize well.

#### 5.1.2. Ensemble ML Models with Default Parameter Input

**Figure 8:**
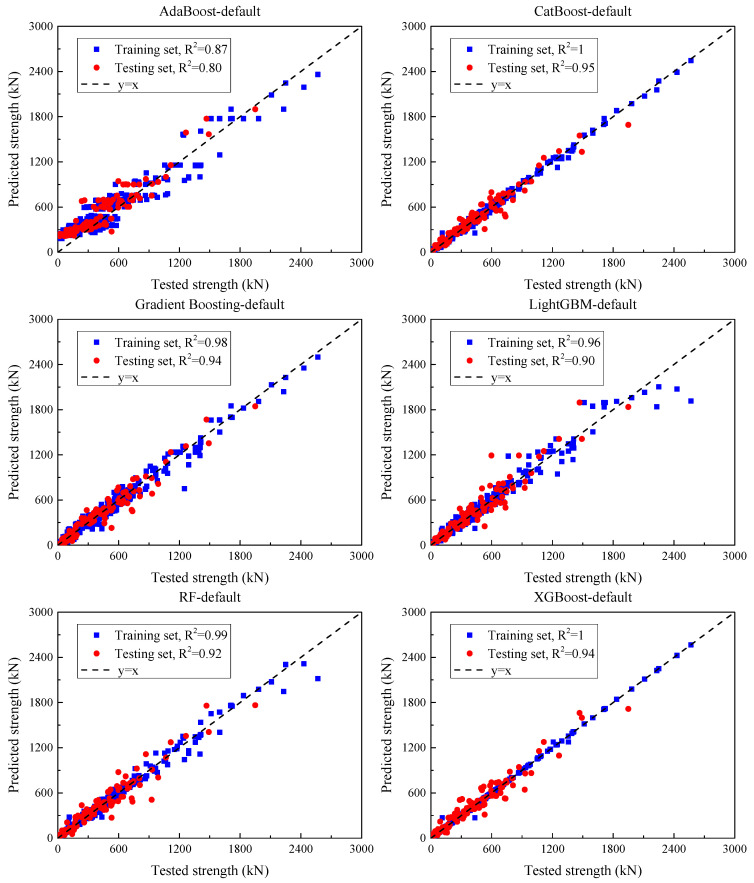
The experimental and predicted SSC by the ensemble models under the default parameter input.

Figure 8 displays the comparison between the experimental and predicted shear strength capacities (SSC) of UHPC beams using ensemble models under default parameter settings. Table 4 summarizes the corresponding performance metrics for both training and test sets.

For AdaBoost, the model recorded an MSE of 21,560.6, an MAE of 124.2, an RMSE of 146.8, and an R^2^ of 0.87 on the training set. However, its performance deteriorated on the test set, where the MSE rose to 22,524.7, MAE reached 123.1, RMSE increased to 150.1, and R^2^ dropped to 0.80, indicating significant overfitting and instability on unseen data.

The GBR model exhibited strong generalization. On the training set, it achieved an MSE of 3407.8, MAE of 39.6, RMSE of 58.4, and R^2^ of 0.98. The test set results—MSE of 7339.3, MAE of 61.1, RMSE of 85.7, and R^2^ of 0.94—demonstrate reliable performance across datasets.

XGBoost displayed excellent learning on the training set, with an extremely low MSE of 166.1, MAE of 4.2, RMSE of 12.9, and a perfect R^2^ of 1.00. On the test set, its MSE rose significantly to 7241.0, MAE to 58.3, RMSE to 85.1, with R^2^ remaining high at 0.94—suggesting strong fitting capability but some overfitting.

LightGBM produced a training MSE of 7095.2, MAE of 39.8, RMSE of 84.2, and R^2^ of 0.96. However, its test set performance declined, with an MSE of 11,949.6, MAE of 67.3, RMSE of 109.3, and R^2^ reduced to 0.90, indicating sensitivity to unseen data.

Random forest (RF) demonstrated solid robustness, achieving a training MSE of 2499.7, MAE of 26.4, RMSE of 50.0, and R^2^ of 0.99. On the test set, its performance remained competitive, with MSE = 9270.8, MAE = 62.8, RMSE = 96.3, and R^2^ = 0.92, highlighting its capacity to balance fit and generalization.

CatBoost outperformed other models overall. It achieved an MSE of 710.7, MAE of 18.6, RMSE of 26.7, and R^2^ of 1.00 on the training set, and it maintained superior performance on the test set with an MSE of 5626.5, MAE of 50.4, RMSE of 75.0, and R^2^ of 0.95—demonstrating strong learning ability and excellent generalization.

In summary, GBR, XGBoost, CatBoost, and RF all exhibited robust performance on both training and test sets. Notably, CatBoost and XGBoost showed exceptionally low training errors, highlighting their strong fitting capacity. However, AdaBoost presented comparatively weaker generalization and was more prone to overfitting.

#### 5.1.3. Deep Learning Models with Default Parameter Input

**Figure 9:**
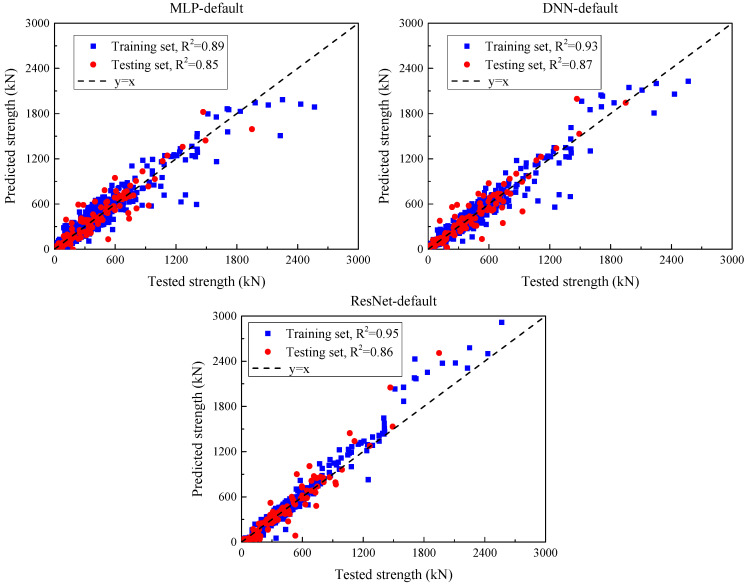
Comparison between predicted and experimental SSC values for each UHPC beam specimen using default deep learning model parameters.

Figure 9 presents a comparison between predicted outputs and actual values obtained from deep learning models using default parameter configurations, and Table 5 summarizes their prediction performance on the training set and test set. The MSE of MLP in the training set is 18568.8, MAE is 88.6, RMSE is 136.3, and R2 is 0.89. These results show that although MLP performs relatively well on the training data, the MSE on the test set is 16964.5, MAE is 9.6, RMSE is 130.2, and R^2^ drops to 0.85, showing a decrease in prediction accuracy on unseen data. The MSE of DNN in the training set is 12472.4, MAE is 68.3, RMSE is 111.7, and R^2^ is 0.92, showing relatively strong fitting performance. However, on the test set, the MSE rose to 14938.8, the MAE increased to 76.7, the RMSE was 122.2, and the R^2^ value dropped to 0.87, showing a certain degree of overfitting. Although DNN performed better than MLP, it still faced similar generalization challenges. ResNet performed well, with an MSE of 9021.1, a MAE of 55.3, a RMSE of 95, and an R^2^ of 0.95 on the training set, indicating that the model was very successful in learning the training data. As for the test set, the MSE was 16592.3, the MAE was 78.3, the RMSE was 128.8, and the R^2^ was 0.86, which was also lower than the previous two but still practical.

### 5.2. Models After Hyperparameter Optimization

To prevent high-variance solutions under grouped k-fold CV, we used capacity-aware hyperparameter bounds (e.g., decision tree max_depth ≤ 10, with min_samples_leaf, feature subsampling, and, for boosting, learning rate/subsample jointly controlling effective complexity). Depth ≥ 10 already yields thousands of partitions, which is sufficient for the present dataset; deeper trees did not provide consistent cross-validated improvements and were thus excluded for parsimony and reproducibility. Table 6 summarizes the hyperparameter optimization of each ML model. Hyperparameters were tuned in two stages: (i) a coarse random/grid search over the ranges in Table 6 (chosen from prior SSC/RC studies and to bound model capacity), followed by (ii) local refinement around the best region. Selection used grouped 5-fold CV with the mean MAE as the primary objective (RMSE as tie-breaker). Early stopping was enabled for gradient-boosting/XGBoost/LightGBM/CatBoost and all deep models. The final values in Table 7 are those minimizing the cross-validated objective.

The optimal parameters of decision tree (DT) include a maximum depth of 10, minimum number of sample splits of 2, and minimum number of leaf samples of 2; random forest (RF) uses 200 trees, a maximum number of features of 0.7, and a maximum depth of 20; AdaBoost and gradient-boosting regression (GBR) are both set to 200 trees, a learning rate of 0.1, and a maximum depth of GRB of 5. XGBoost and LightGBM both use 600 trees; the former has a maximum depth of 4, and the latter has a learning rate of 0.2; CatBoost also uses 400 iterations and a learning rate of 0.1. The optimal structure of multilayer perceptron (MLP) is a hidden layer configuration of (100, 50, 25), combined with the relUactivation function and a learning rate of 0.1; deep neural network (DNN) is set to 3 layers, the number of initial units is 256, etc. Finally, residual network (ResNet) has 160 initial units and 5 blocks, and the learning rate is 0.00265.

#### 5.2.1. Single ML Models After Hyperparameter Optimization

**Figure 10:**
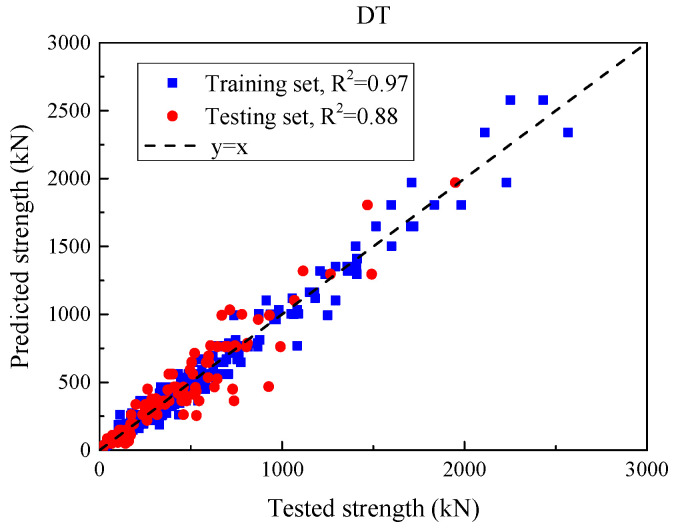
Comparison of DT model prediction values and experimental values for each beam specimen after hyperparameter optimization.

Figure 10 and Table 7 show that, even after tuning, the DT attains near-perfect in-sample fit (train: MSE = 4462.2 kN^2^, MAE = 40.7 kN, RMSE = 66.8 kN, R^2^ = 0.97) but degrades on the hold-out set (test: MSE = 13,621.9 kN^2^, MAE = 75.8 kN, RMSE = 116.7 kN, R^2^ = 0.88). This discrepancy arises from two well-known factors. (i) Model variance. A single decision tree produces piecewise-constant partitions and tends to memorize training patterns when depth is sufficient; small perturbations in the data (or a different split) can change leaf boundaries and inflate test errors—a behavior frequently reported for structural SSC datasets. (ii) Dataset heterogeneity and scale. Our database aggregates tests from multiple sources with different section types and ranges of geometry, reinforcement, and loading; such a covariate shift between the train and test subsets (e.g., more high-capacity members in the hold-out) increases absolute-error metrics (MAE/RMSE) even when the rank correlation captured by R^2^ remains high—an observation consistent with broader RC studies that benchmark ML using error-based criteria and cross-validation.

Using grouped 5-fold cross-validation—with groups defined by the publication/test program to prevent cross-study leakage—the DT achieved MSE = 15,885.1 ± 3177.0 kN^2^, RMSE = 126.0 ± 12.6 kN, MAE = 81.9 ± 8.2 kN, and R^2^ = 0.86 ± 0.03 (mean ± SD across folds; Table 8). Compared with the single 80/20 hold-out baseline (RMSE = 116.7 kN, MAE = 75.8 kN, R2 = 0.88), the cross-validated scores are slightly more conservative, as expected under a stricter evaluation protocol that keeps correlated specimens in the same fold. The fold-to-fold dispersion is modest (e.g., SD ≈ 10% of RMSE), indicating stable generalization of the decision tree across partitions and providing a reliable baseline for comparison with more expressive models.

#### 5.2.2. Ensemble ML Models

**Figure 11:**
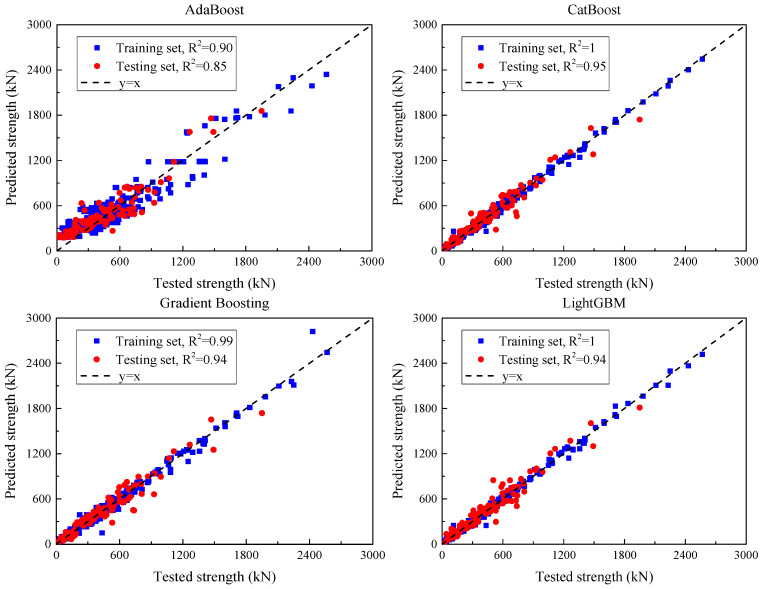

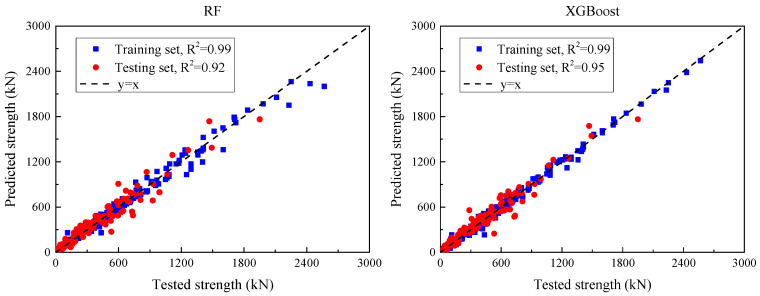
Comparison of ensemble model predictions with experimental shear strengths for UHPC beam specimens after hyperparameter tuning.

Figure 11 illustrates the predicted versus observed values of each ensemble model after hyperparameter tuning, while Table 9 summarizes their predictive performance on the training and test sets.

For AdaBoost, the training set results yielded an MSE of 17,583, an MAE of 108.0, and an R^2^ of 0.90. On the test set, the MSE was 17,276.5, the MAE 108.5, and the R^2^ dropped to 0.85. Despite the acceptable R^2^, the relatively large errors suggest that AdaBoost exhibits limited fitting capacity and lacks robustness on unseen data.

In contrast, gradient boosting regression (GBR) demonstrated excellent learning performance, with an MSE of 1844.9, an MAE of 28.0, and an R^2^ approaching 1.00 on the training set. On the test set, the MSE increased to 6906.9 and the MAE to 57.3, with an R^2^ of 0.94, indicating strong generalization capability.

XGBoost also showed outstanding performance, achieving an MSE of 888.6, an MAE of 20.5, and an R^2^ of 0.99 on the training set. On the test set, the MSE and MAE rose to 6248.7 and 53.1, respectively, with an R^2^ of 0.95, demonstrating high accuracy and robustness in handling complex nonlinear relationships.

LightGBM produced similar results to XGBoost, with an MSE of 686.5, an MAE of 16.5, and an R^2^ of 0.99 on the training data. Its test set performance included an MSE of 6532.2, MAE of 55.1, and R^2^ of 0.94, confirming both accuracy and stability.

Random forest (RF) achieved an MSE of 2287.2, MAE of 25.1, and R^2^ of 0.99 on the training set, with corresponding test set values of 7646.5 (MSE), 60.1 (MAE), and 0.93 (R^2^). These results confirm RF’s strong adaptability and competitive predictive capability.

Finally, CatBoost outperformed all other models. On the training set, it attained an MSE of 780.6, an MAE of 19.7, and a perfect R^2^ of 1.00. On the test set, it maintained excellent performance, with an MSE of 5680.9, MAE of 50.9, and R^2^ of 0.95, indicating superior generalization and precision.

In summary, GBR, XGBoost, LightGBM, and CatBoost exhibited strong predictive capabilities for UHPC shear strength estimation, with CatBoost demonstrating the most consistent performance across both datasets. Conversely, AdaBoost lagged behind in accuracy and generalization, highlighting the need for further enhancement.

Using grouped 5-fold cross-validation—with groups defined by the publication/test program to prevent cross-study leakage—Table 10 summarizes the generalization of six ensemble models (means ± SD across folds). CatBoost achieves the best accuracy (RMSE = 81.4 ± 8.1 kN; MAE = 55.0 ± 5.5 kN; R^2^ = 0.94 ± 0.012), closely followed by XGBoost (85.3 ± 8.5 kN; R^2^ = 0.93 ± 0.014) and LightGBM (87.3 ± 8.7 kN; R^2^ = 0.93 ± 0.014). GBR and RF perform slightly worse (GBR: 89.7 ± 9.0 kN; R^2^ = 0.91 ± 0.018; RF: 94.4 ± 9.4 kN; R^2^ = 0.92 ± 0.016), while AdaBoost lags behind (141.9 ± 14.2 kN; R^2^ = 0.84 ± 0.032). Fold-to-fold dispersion is modest (RMSE SD ≈ 9–10% of the mean), indicating a stable ranking across partitions. Relative to the single 80/20 hold-out (reported elsewhere), cross-validated errors are slightly larger and R^2^ slightly lower (≈ +7–10% RMSE; −0.01 to −0.03 in R^2^), as expected under the stricter grouped protocol.

#### 5.2.3. Deep Learning Models After Hyperparameter Optimization

**Figure 12:**
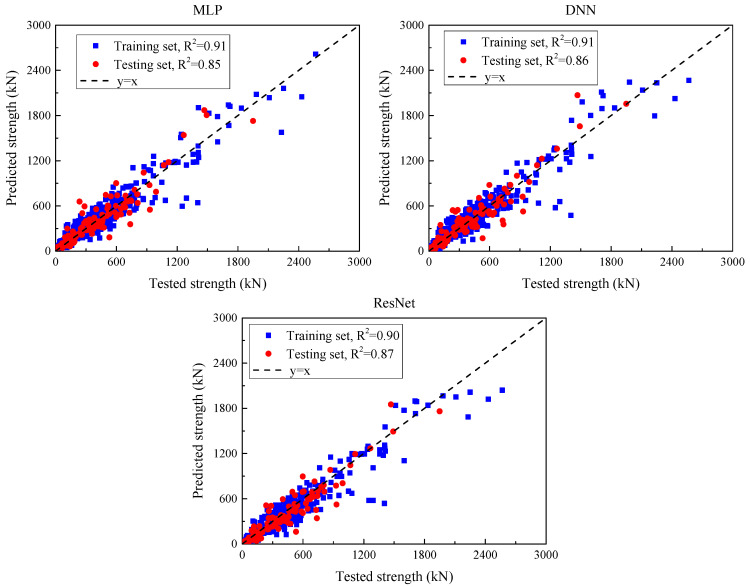
Comparison of predicted values of the deep learning model and experimental values of each beam specimen after hyperparameter optimization.

Figure 12 presents a comparison between the predicted and observed values of the deep learning models after hyperparameter tuning, while Table 11 summarizes their predictive performance across both training and test sets. For the MLP model, the MSE on the training set is 15,386.6, while it rises to 16,853.7 on the test set, indicating a strong fit to training data but reduced generalization. The corresponding MAE values are 80.2 and 87.8, and the RMSE values are 124.0 and 129.8 for the training and test sets, respectively. These metrics reflect a noticeable prediction deviation and error spread. The R^2^ is 0.91 for the training set and 0.85 for the test set, demonstrating reasonable predictive accuracy but evident overfitting. The DNN model exhibits similar behavior. It records an MSE of 15,413.1 on the training set and 16,257.1 on the test set, with corresponding MAE values of 77.2 and 81.4. The RMSE values are 124.1 (training) and 127.5 (test). R^2^ remains consistent with MLP, at 0.91 and 0.85, respectively. This indicates that while DNN slightly outperforms MLP in error magnitude, it still suffers from limited generalization capacity. In contrast, ResNet yields an MSE of 17,879.9 on the training set and 14,707.3 on the test set, indicating relatively better generalization. MAE is 82.9 on the training set and 84.6 on the test set, while RMSE values are 121.3 and 127.5, suggesting improved robustness in prediction. The R^2^ values are 0.90 (training) and 0.87 (test), indicating stable predictive accuracy across both sets, though a moderate overfitting trend is still observed.

In summary, all three deep learning models demonstrate strong learning capabilities on training data; however, performance degradation on the test set suggests varying degrees of overfitting. Among them, ResNet shows slightly better generalization, while MLP and DNN offer lower training error but less stable test performance.

Under grouped 5-fold cross-validation—groups defined by publication/test program to preclude cross-study leakage—three deep models exhibit moderate and internally consistent accuracy (Table 12; means ± SD across folds; RMSE/MAE in kN, MSE in kN^2^). ResNet performs best among the deep learners (RMSE = 131.0 ± 13.1 kN; MAE = 91.4 ± 9.1 kN; R^2^ = 0.85 ± 0.030), followed by DNN (137.7 ± 13.8 kN; R^2^ = 0.83 ± 0.034) and MLP (140.2 ± 14.0 kN; R^2^ = 0.82 ± 0.034). Fold-to-fold dispersion remains modest (RMSE SD ≈ 9–10% of the mean), indicating stable generalization across partitions. Compared with the tree-based gradient-boosting models (Table 11), the deep networks yield higher errors and lower R^2^, which is consistent with tabular, medium-scale datasets where boosted trees typically capture nonlinear interactions and thresholds more efficiently.

### 5.3. Comparison

**Figure 13:**
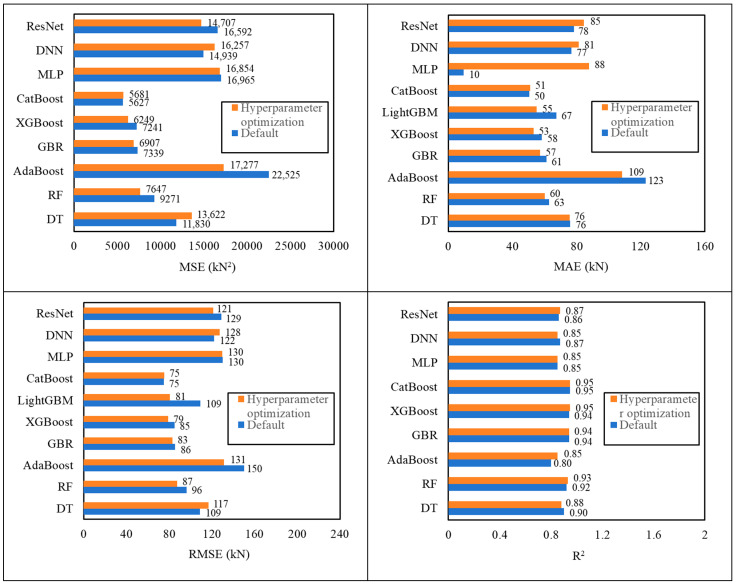
Comparison of predicted values and experimental values of each machine learning model after hyperparameter optimization.

Figure 13 summarizes the prediction ability evaluation of each model on the test dataset before and after hyperparameter optimization. When comparing the model performance before and after hyperparameter optimization, it can be found that the performance of most models is significantly improved, especially the generalization ability on the test set. DT performed extremely well in the training set but showed obvious overfitting in the test set. Although it improved after optimization, it still did not reach the ideal level. The performance of the integrated model AdaBoost is slightly improved after optimization, but the overall performance is still insufficient. In contrast, GBR, XGBoost, and LightGBM show stability and superiority. After optimization, GBR continues to perform well on the training and test sets and is not affected by overfitting; XGBoost and LightGBM also perform well in the MSE and R^2^ indicators on the test set, indicating that these algorithms have excellent learning capabilities for data. Although the performance of RF on the test set slightly decreased, it still remains at a high level, showing its robustness. CatBoost performs excellently under default settings, and hyperparameter tuning produces only negligible changes on the test set (Figure 13), indicating high robustness to tuning. In contrast, although the performance of deep learning models (such as MLP, DNN, and ResNet) on the training set improved, the MSE on the test set is high and fails to improve significantly, suggesting that these models may have overfitting problems.

Overall, hyperparameter optimization significantly improves most tree-based ensembles—particularly XGBoost and LightGBM—with moderate gains for AdaBoost/RF/DT and GBR. CatBoost does not materially benefit from tuning on our dataset and can be used with default or lightly tuned settings.

## 6. Model Stacking Based on the Predictive Capabilities of Each ML Model

There are obvious differences in the sensitivity of each machine learning model to different characteristic data. Based on MSE, MAE, RMSE and R^2^, each model is weighted, and a UHPC beam shear capacity prediction model is established by weighted averaging. This method can combine the advantages of multiple models and fully reflect their respective response capabilities to different characteristic data, thereby producing more accurate prediction results. Integrating the outputs of each model by weighted averaging can effectively reduce the error of a single model and improve the robustness and reliability of the overall prediction. This weighting strategy enables the final prediction model to not only reflect the influence of multiple data characteristics but also has strong adaptability, good effectiveness, and accuracy.

Four models with better prediction performance for the test set are selected from the above machine learning models, GBR, XGBoost, LightGBM, and CatBoost. Each model is weighted by MSE, MAE, RMSE, and R^2^, respectively, as *w*_1*i*_, *w*_2*i*_, *w*_3*i*_, and *w*_4*i*_, as shown in the following formula, where *i* represents the weight of the *i*-th model.

(1) $$\left\{\begin{array}{l} w_{1i}=\frac{\frac{1}{MSE_{i}}}{\sum_{n=1}^{4}\frac{1}{MSE_{n}}}\cdot \frac{1}{4},w_{2i}=\frac{\frac{1}{MAE_{i}}}{\sum_{n=1}^{4}\frac{1}{MAE_{n}}}\cdot \frac{1}{4}\\ w_{3i}=\frac{\frac{1}{RMSE_{i}}}{\sum_{n=1}^{4}\frac{1}{RMSE_{n}}}\cdot \frac{1}{4},w_{4i}=\frac{R_{i}^{2}}{\sum_{n=1}^{4}R_{n}^{2}}\cdot \frac{1}{4} \end{array}\right.$$

Then, the weight of the *i*-th model is as follows:

(2) $$w_{i}=w_{1i}+w_{2i}+w_{3i}+w_{4i}$$

The calculated shear bearing capacity of UHPC after weighted averaging is as follows:

(3) $$V_{n}=\sum_{i=1}^{4}w_{i}V_{i}$$

**Figure 14:**
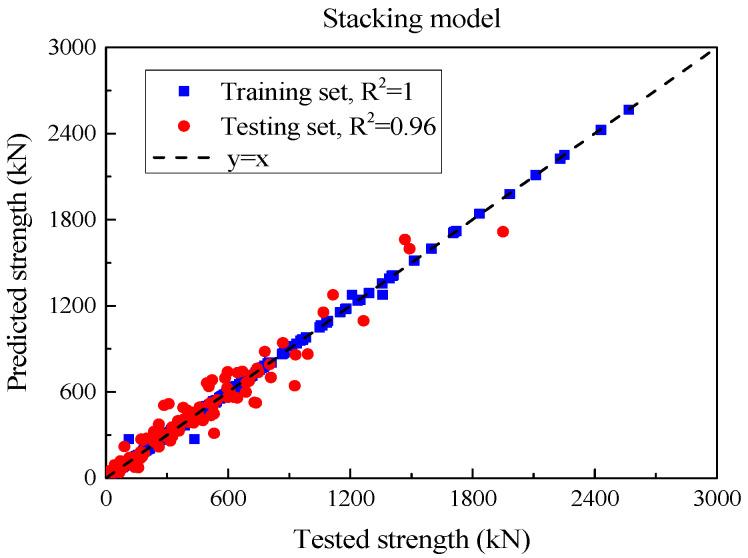
Comparison of stacking model predictions with experimental shear strength values.

The SSC of each UHPC beam was obtained by the above method and compared with the experimental value, as shown in Figure 14. Table 13 summarizes the performance of each model in the test set. From an MSE point of view, *V_n_* significantly reduced the MSE to 4843.3 after using the weighted average method, highlighting that it is superior to all individual models in prediction accuracy. RMSE also reflects this trend. CatBoost’s RMSE is 75.4, XGBoost and LightGBM are 79.0 and 80.8 respectively, while *V_n_*’s RMSE is only 69.6, further proving its lower prediction error. In terms of MAE, CatBoost is 50.9, while XGBoost and LightGBM have MAEs of 53.1 and 55.1, respectively. However, the MAE of *V_n_* is further reduced to 47.0, demonstrating the excellent performance of the model. This performance improvement is not only reflected in the error index, as *V_n_* also performs well in R^2^ (coefficient of determination), with an R^2^ of 0.96, which exceeds CatBoost and XGBoost’s 0.95, showing that it has a stronger ability to explain data variation. The coefficient of variation (CoV) of the ratio of calculated values to experimental values is a low value of 0.17, indicating that the prediction results of *V_n_* have higher stability and consistency. Compared with the CoV of GBR, XGBoost and LightGBM (0.23, 0.24, and 0.30 respectively), *V_n_* ’s performance is more reliable. In terms of mean ratio, the values of each model are close to 1, indicating that the prediction of shear bearing capacity is relatively reasonable. The mean ratio of *V_n_* is 1.05, which is slightly higher than that of other models, indicating that its predicted value is slightly higher than the experimental value. In general, as a comprehensive model, *V_n_* effectively integrates the advantages of other models by weighted averaging, showing obvious prediction advantages. In terms of multiple key performance indicators, *V_n_* not only leads the single model but also demonstrates improved overall performance in terms of accuracy, explanatory power, and output stability.

## 7. The 95% Confidence Interval

Multiple machine learning models (GBR, XGBoost, CatBoost, and LightGBM) are used to predict the SSC of UHPC beams, and weights are assigned according to the MSE, MAE, RMSE, and R^2^ of each model, and then, a 95% confidence interval is established through the residual. First, by integrating the prediction results of multiple models, the advantages of each model under different data characteristics can be fully utilized, thereby improving the robustness and accuracy of the overall prediction and offsetting the limitations of a single model. At the same time, the use of residuals to calculate confidence intervals provides users with a quantification of prediction uncertainty, which can effectively reduce risks in the decision-making process. Secondly, this method performs well in improving interpretability. By displaying confidence intervals, the credibility and potential errors of model predictions are more clearly displayed. At the same time, the introduction of confidence intervals enhances risk management capabilities, allowing decision makers to more comprehensively evaluate prediction risks and reduce losses caused by prediction bias.

The residual, representing the error between predicted and observed values for each model, is computed using the following equation:

(4) $$e_{i}=V_{ui}-V_{ni}$$

where *V_ui_* is the shear bearing capacity test value of the *i*-th UHPC beam specimen, and *V_ni_* is the weighted average shear bearing capacity of the *i*-th UHPC beam specimen.

**Figure 15:**
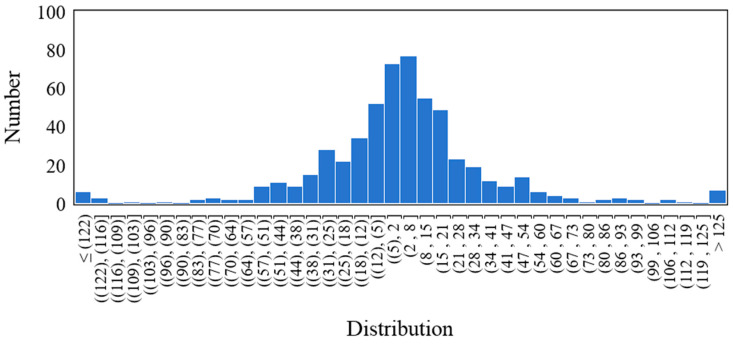
Residual distribution.

**Figure 16:**
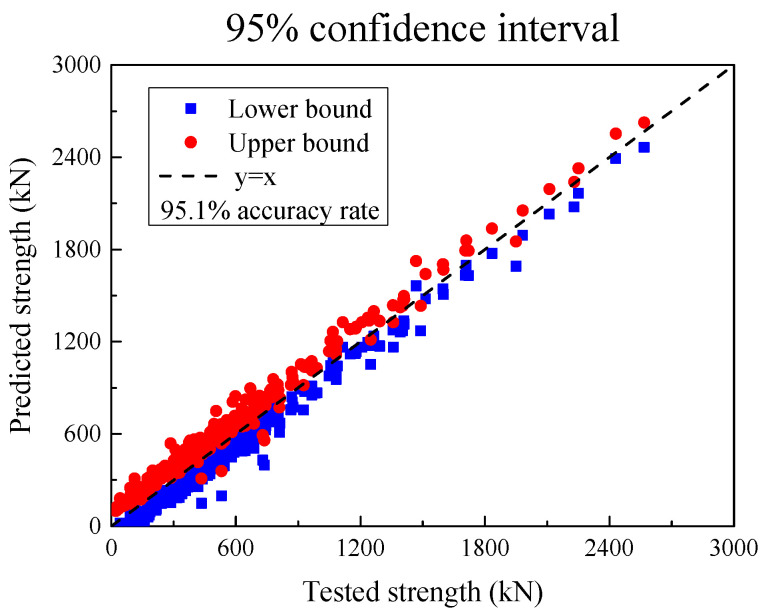
The 95% confidence interval.

The residual distribution of each UHPC beam specimen obtained above is shown in Figure 15 below. The histogram of out-of-fold residuals is bell-shaped and centered near zero, which is consistent with an approximately normal, zero-mean error distribution. We note that a single histogram cannot fully diagnose heteroscedasticity or dependence; therefore, we also report the empirical coverage of the 95% intervals shown as Figure 16, which is close to the nominal level, and it supports the adequacy of the normal approximation for our data.

This gives the standard deviation of the weighted residual:

(5) $$\sigma =\sqrt{\frac{1}{n-1}\sum_{i=1}^{n}{\left(e_{i}-\bar{e}\right)}^{2}}$$

where $\bar{e}$ is the mean value of the residuals, and the 95% confidence interval can be expressed as

(6) $$CI=V_{n}\pm 1.96\sigma$$

where 1.96 means that under a normal distribution, the z value corresponding to the 95% confidence interval is approximately 1.96.

The SSC of each UHPC beam is shown below, with red as the upper limit and blue as the lower limit. For the entire data set, 95.1% of the shear bearing capacity test values of the UHPC beam specimens are within this interval.

## 8. Simplified Calculation Method for Engineering Design

### 8.1. Simplified Calculation Method

The SSC of UHPC beams is affected by many factors, and there is no unified shear failure mechanism and shear capacity formula. There are large differences between the current design specifications (such as French NF P18-710 [106], Swiss SIA 2052 [107], Japanese JSCE-2006 [108], GB 50010-2010 [109], CECS 38:2004 [110], etc.), so it is urgent to conduct a systematic study on the shear performance and bearing capacity calculation method of UHPC beams. To this end, based on the UHPC beam shear test database with a wider range of parameters, the bearing capacity calculation formula in the current specifications is evaluated so as to revise and simplify the relevant formulas of the NF P18-710 specification and the CECS 38:2004. The SSC formulas of UHPC beams in French NF P18-710, Swiss SIA 2052, Japanese JSCE-2006, GB 50010-2010, CECS 38:2004, and other regulations are as follows:

(1) French NF P 18-710 standard [106].

(7) $$V_{u}=\frac{0.21}{\gamma_{cf}\gamma_{E}}k\sqrt{f_{c}}bh_{o}+\frac{A_{s}}{s}zf_{sv}\cot\theta +A_{fv}\sigma_{f}\cot\theta$$

where *γ_cf_*, *γ_E_* are the safety factors, which are taken as 1.0 when analyzing the test data in this paper; *k* is the prestress influence coefficient, which is taken as 1.0 when there is no prestress; *θ* is the angle between the principal compressive stress and the horizontal direction, which can be taken as 45° for reinforced UHPC; *A_fv_* is the area of fiber action on the inclined crack, which can be taken as *bz*; and *σ_f_* is the residual tensile strength of UHPC.

(2) Swiss SIA 2052 guidelines [107].

(8) $$V_{u}=\frac{bz\cdot 0.5(f_{Uted}+f_{Utud})}{\tan\alpha }+\frac{A_{s}}{s}\cdot z\cdot f_{sv}(\cot\alpha +\cot\beta )\sin\beta$$

where *f_Uted_* and *f_Utud_* are the first crack strength and ultimate tensile strength of UHPC, respectively. For the convenience of calculation, this paper uniformly takes the axial tensile strength of UHPC *f_t_*; *α* and *β* are the angles between the principal compressive stress and the principal tensile stress and the beam axis direction, respectively.

(3) Japanese JSCE-2006 guidelines [108].

(9) $$V_{u}=0.18\sqrt{f_{c}}bh_{o}/\gamma_{b}+\left(\sigma_{f}/\tan\theta \right)bz/\gamma_{b}+P_{e}\sin\alpha_{p}/\gamma_{b}$$

where *V_p_* is the shear bearing capacity of the prestressed tendon; *θ* is the angle between the component axis and the oblique crack; *P_e_* is the tension force of the prestressed tendon; *α_p_* is the angle between the prestressed tendon and the horizontal direction; and *γ_b_* is the partial coefficient, which is taken as 1.0.

(4) GB 50010-2010 [109].

(10) $$V_{u}=\frac{1.75}{\lambda +1}f_{t}bh_{o}+f_{sv}\cdot \frac{A_{s}}{s}\cdot h_{o}+0.05N_{po}$$

*λ* is the shear span ratio. When *λ* < 1.5, *λ* = 1.5; when *λ* > 3.0, *λ* = 3.0; *f_t_* is the tensile strength of concrete; and *N_po_* is the tension force of the prestressed tendon.

(5) CECS 38:2004 [110].

(11) $$V_{u}=\frac{1.75}{\lambda +1}(1+\beta_{v}\lambda_{f})f_{t}bh_{o}+f_{sv}\cdot \frac{A_{s}}{s}\cdot h_{o}+0.05N_{po}$$

where *β_v_* is 0.45, and *λ_f_* = *ρ_f_l_f_*/*d_f_*.

Based on the formulation structure provided in CECS 38:2004 and utilizing the established experimental dataset, a revised empirical equation for the shear capacity of UHPC beams is proposed in Equation (12):

(12) $$V_{u}=\beta_{1}X_{1}+\beta_{2}X_{2}+\beta_{3}X_{3}+\beta_{4}X_{4}$$

In Equation (12), each bracketed expression (*X_i_*) is treated as a single fitted regressor; the coefficients *β_j_* are estimated against these composite variables. The following is defined:

(13) $$X_{1}=\frac{f_{t}bh_{o}}{\lambda +1},X_{2}=\frac{\lambda_{f}f_{t}bh_{o}}{\lambda +1},X_{3}=f_{sv}\cdot \frac{A_{s}}{s}\cdot h_{o},X_{4}=N_{po}$$

with the following coefficients (calibrated on our database):

(14) $$\left\{\begin{array}{l} \beta_{1}=3.72\\ \beta_{2}=3.18\\ \beta_{3}=1.05\\ \beta_{4}=0.069 \end{array}\right.$$

**Figure 17:**
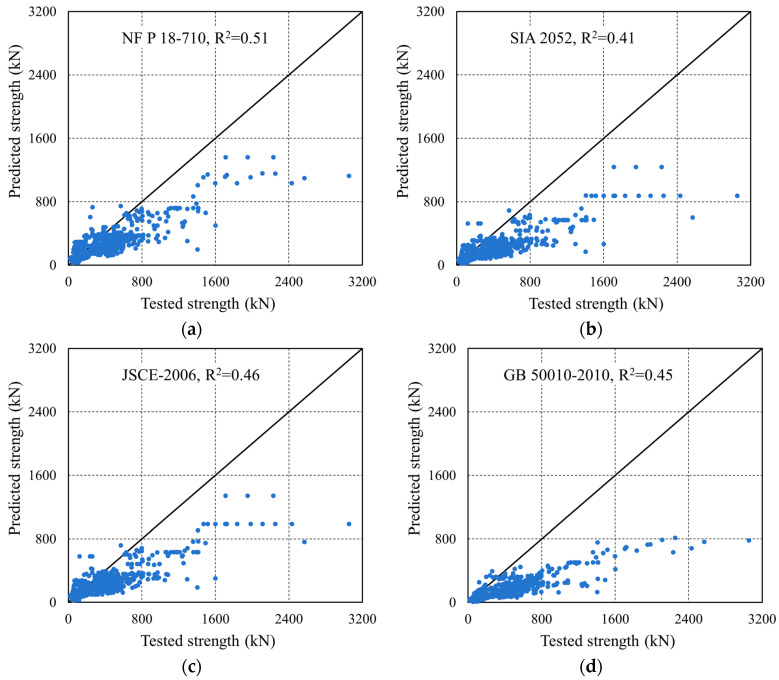

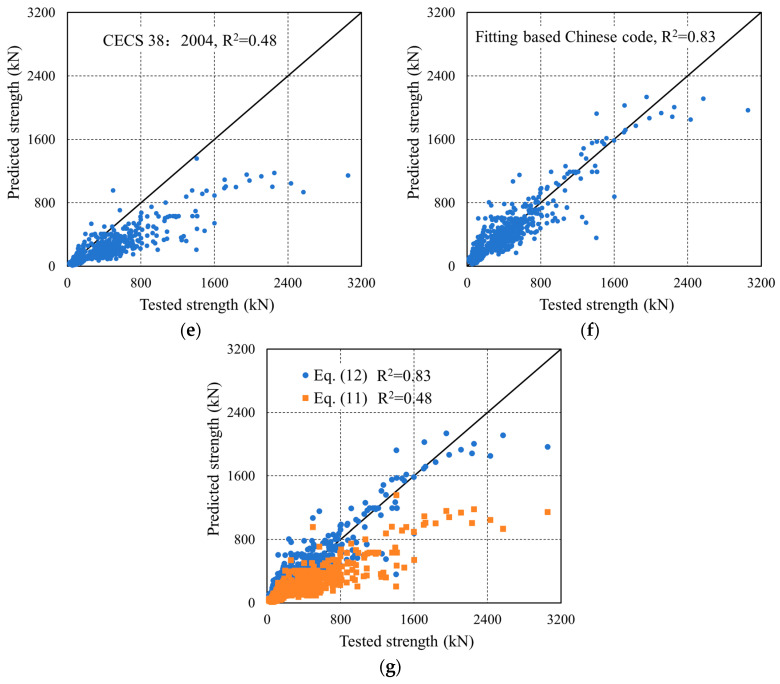
Comparison between theoretical calculation model prediction value and experimental value: (**a**) French NF P18-710; (**b**) Swiss SIA 2052; (**c**) Japanese JSCE-2006; (**d**) GB 50010-2010; (**e**) CECS 38:2004; (**f**) Fitting equation of Equation (12); (**g**) Comparison between Equations (11) and (12).

Figure 17 illustrates the comparison between the predicted and experimental SSC of UHPC beams obtained using different methods, and Table 12 shows the MAE, MSE, RMSE, R^2^ and other values of each calculation method.

The numerical comparison shows that the proposed data-calibrated equation achieves higher accuracy (e.g., higher R^2^ with lower MAE/RMSE, presented in Table 14) than several reference formulas; however, the reasons for the gap are structural.

- Scope and calibration domain. Some provisions were not calibrated on UHPC beams or only on narrow ranges of fiber content, reinforcement, and shear span ratios. When applied to our wider dataset—covering rectangular/T/I sections and various a/d—systematic bias appears.
- Missing or aggregated mechanisms. Several formulas do not include an explicit fiber term or do not capture fiber–stirrup interactions, size, and a/d effects with sufficient fidelity, leading to under- or over-predictions when fibers control crack bridging or when transverse steel is sparse/dense.
- Design format vs. mean prediction. Code expressions often target characteristic resistances with partial safety factors, whereas our benchmarking uses mean test values; this intentional conservatism can manifest as weaker predictive statistics (larger MAE/RMSE, lower R^2^), even when the provision is appropriate for design safety.

Compared with standard code formulas, the proposed equation (Equation (12)) is spreadsheet-ready and uses the same design-level inputs. It provides, in addition to a point estimate, prediction intervals derived from residuals, enabling reliability-aware choices (e.g., using a one-sided lower bound or a resistance factor ϕ). In practice, this (i) reduces unnecessary conservatism where code formulas are markedly biased for UHPC beams, (ii) keeps implementation cost negligible (single-cell formula, no specialized software), and (iii) remains compatible with code checks, functioning as a companion tool rather than a replacement. For cases outside the data domain, conservative usage (interval lower bound or smaller ϕ) is recommended.

Overall, the performance differences across provisions are consistent with calibration scope and mechanism coverage, while the proposed equation offers a practical, low-cost supplement that improves accuracy and provides transparent uncertainty information for engineering decisions.

### 8.2. Implications for Engineering Practice

This study offers two complementary deliverables for the design and assessment of UHPC beams in shear:

(1) Code-compatible closed-form design equation.

The proposed formula (Equation (12)) is expressed as a sum-of-force-type regressor with dimensionless coefficients, returning *V*_*u*_ in kN from inputs in MPa/mm. It is spreadsheet-ready, requires only standard design variables (geometry, *f*_*c*_, fiber factor, transverse steel) and is aligned with common code layouts (shear span factor, stirrup term, axial effect). Engineers can thus use it directly for preliminary sizing, option screening, and rapid checks alongside code provisions.

(2) Reliability-aware verification via ML + intervals.

The cross-validated ensemble (boosted trees/stack) supplies a point estimate and a 95% prediction interval (PI) derived from residuals. Two practical uses follow:

Safety margin check: it accepts designs for which the demand *V*_Ed_ is below the 95% lower bound *L* = *V*_n_ − 1.645 σ, with an engineering margin (e.g., *V*_Ed_ ≤ 0.9 *L*).

Conservative design value: when a single number is required, it adopts *Vd* = *ϕV*_*u*_, with *ϕ* chosen to at least remove mean bias (e.g., *ϕ* ≈ 0.95), or takes *Vd* = *L* for a one-sided 95% design.

(3) Where it helps most.

- Fiber–stirrup trade-off: the explicit terms in Equation (12) and feature importance from ML highlight how the stirrup spacing *s* and fiber content *ρ*_*f*_ jointly influence capacity, informing economical mixes of transverse steel and fibers in short-span/low-height members.
- Assessment/retrofit: for existing members with measured properties, the model provides an unbiased capacity estimate with quantified uncertainty, aiding rating decisions and retrofit prioritization.
- Parametric exploration: rapid “what-if” scans (e.g., changing a/d, *ρsv*, *ρf*) identify efficient regions before detailed nonlinear analysis.

(4) Applicability domain.

The formula and ML models are calibrated over the database ranges reported in the paper. For inputs outside these ranges, (i) it flags as extrapolation, (ii) relies on the lower PI bound or a smaller *ϕ*, and (iii) corroborates with mechanics-based checks. Before use, it ensures unit consistency (MPa, mm, kN) and that composite regressors (bracketed terms) are computed as kN.

(5) Workflow for practice.

Step 1: Use Equation (12) for a first estimate of *V*_*u*_ (kN).

Step 2: Perform code checks required by the governing standard.

Step 3: Verify with the ML predictor and extract the 95% PI.

Step 4: Select a conservative design value via *Vd* = *ϕVu* (e.g., *ϕ* ≈ 0.95) or the PI lower bound; document the choice and the input ranges.

In summary, the closed-form equation supports fast, code-compatible estimation, while the ML + PI verification adds a transparent, reliability-aware layer for critical design decisions.

While this work targets shear resistance, design should also check rotation capacity in the prospective plastic-hinge region. Recent analyses indicate that the available plastic rotation depends on the plasticization length and boundary restraint and that insufficient ductility can govern ultimate capacity via premature (non-fully developed) mechanisms. In UHPC members, the longitudinal reinforcement ratio/detailing, transverse reinforcement (stirrups), and fiber bridging jointly influence crack control, confinement, hinge length, and thus, rotation capacity for redistribution. We therefore flag ductility verification as a companion check to shear and plan a follow-up study that curates rotation-capacity indicators and develops uncertainty-aware predictors under the same grouped-CV protocol [111,112].

## 9. Conclusions

This study presents a practice-oriented framework for estimating the shear strength of UHPC beams by combining grouped k-fold cross-validated machine-learning models, a residual-based treatment that provides prediction intervals, and a spreadsheet-ready design equation formulated with force-type regressors (kN) and dimensionless coefficients. The framework is transparent and compatible with common code variables and is intended to complement mechanics-based checks in preliminary sizing, verification, and assessment. The approach clarifies the roles of geometry, matrix strength, fibers, and transverse reinforcement while communicating uncertainty alongside point predictions, thus supporting accountable decision-making in design and evaluation.

### Limitations and Outlook

This study prioritizes a stacking ensemble with residual-based intervals calibrated via grouped k-fold CV. While Bayesian regression and Gaussian-process regressors are standard tools for predictive uncertainty, a rigorous head-to-head evaluation would entail non-trivial prior/kernel specification and inference choices under nested grouped CV, which lies beyond the present scope.

Future work will focus on multi-laboratory blind validation across slabs, deep beams, joints, and prestressed members; reliability-based calibration of resistance factors for direct design adoption; richer uncertainty modeling (heteroscedastic, quantile/empirical, and Bayesian/GP within the same grouped-CV protocol); incorporation of physics-informed features to strengthen extrapolation; and reproducible data/model releases to enable periodic updates and broader deployment in practice.

## Figures and Tables

**Table 1:** Details of newly curated UHPC beam studies (this paper).

No.	Year	References	Specimen No.	Types	Main Test Parameters
1	2025	Hao et al. [101]	5	T-R-S	λ, tw1
2	2025	Jin et al. [102]	11	REC-R-S	Vf, with/without stirrups
3	2023	Chen et al. [103]	11	REC-R-S	Vf
4	2024	Zhang et al. [104]	6	REC-R-S	Vf
5	2024	Frank et al. [105]	6	REC-R-S	Vf,ρf

Note: Only studies added by this work are listed here; the 523 specimens from Ye et al. [23] are cited as an aggregated entry. T/REC represent T-shaped and rectangular cross-sections, respectively, R represents reinforced, and S represents stirrups.

**Table 2:** UHPC beam shear bearing capacity test database data characteristics statistics.

Parameter	*h* (mm)	*b* (mm)	*b*_f1_ (mm)	*t*_f1_ (mm)	*b*_f2_ (mm)	*t*_f2_ (mm)	*A*_c_ (mm^2^)	*ρ*_l_ (%)	*f*_sy_ (MPa)	*ρ*_p_ (%)	*σ*_p_ (MPa)	*s* (mm)	*ρ*_sv_ (%)	*f*_sv_ (MPa)	*f*_c_ (MPa)	*ρ*_f_ (%)	*l*_f_ (mm)	*d*_f_ (mm)	*m*	*V*_u_ (kN)
mean	361	107	143	32	95	42	54045	0.052	444	0.009	2.90	57	0.002	156	143	1.5	14	0.22	2.6	433.1
std	194	53	184	39	150	60	42769	0.046	245	0.017	6.09	97	0.004	230	26	0.8	10	0.16	1.0	400.5
min	76	20	0	0	0	0	8400	0.000	0	0.000	0.00	0	0.000	0	78	0.0	0	0.00	0.6	18.2
25%	240	60	0	0	0	0	27250	0.021	400	0.000	0.00	0	0.000	0	123	1.0	13	0.20	1.8	151.9
50%	330	100	0	0	0	0	43400	0.044	481	0.000	0.00	0	0.000	0	138	1.6	13	0.20	2.5	361.7
75%	400	150	250	60	165	80	70000	0.071	548	0.012	0.00	100	0.003	423	161	2.0	13	0.20	3.5	528.5
max	1092	250	737	190	737	228	276939	0.223	1100	0.074	27.07	500	0.021	731	224	5.0	60	1.00	8.0	3053.0

Note: *b*_f1_/*t*_f1_ (top flange) and *b*_f2_/*t*_f2_ (bottom flange) are in mm; paired zeros denote that the corresponding flange is absent.

**Table 3:** Prediction performance of DT model under default parameter input.

Models		MSE (kN^2^)		MAE (kN)		RMSE (kN)		R^2^	
		Train	Test	Train	Test	Train	Test	Train	Test
Single	DT	148.3	11,829.6	1.5	76	12.2	108.8	1	0.9

**Table 4:** Prediction performance of each ensemble model under default parameter input.

Models		MSE (kN^2^)		MAE (kN)		RMSE (kN)		R^2^	
		Train	Test	Train	Test	Train	Test	Train	Test
Ensemble	AdaBoost	21560.6	22524.7	124.2	123.1	146.8	150.1	0.87	0.8
	GBR	3407.8	7339.3	39.6	61.1	58.4	85.7	0.98	0.94
	XGBoost	166.1	7241	4.2	58.3	12.9	85.1	1	0.94
	LightGBM	7095.2	11949.6	39.8	67.3	84.2	109.3	0.96	0.9
	RF	2499.7	9270.8	26.4	62.8	50	96.3	0.99	0.92
	CatBoost	710.7	5626.5	18.6	50.4	26.7	75	1	0.95

Note: Color legend (based on test metrics only): green (meets) R^2^ ≥ 0.93, MAE ≤ 60 kN, RMSE ≤ 90 kN; yellow (marginal) R^2^ ≥ 0.90, MAE ≤ 90 kN, RMSE ≤ 110 kN; red (below) otherwise. The last column reports the accuracy band with text labels for accessibility; train columns are not used for acceptance.

**Table 5:** Prediction performance of each deep learning model under default parameter input.

Models		MSE (kN^2^)		MAE (kN)		RMSE (kN)		R^2^	
		Train	Test	Train	Test	Train	Test	Train	Test
Deep learning	MLP	18568.8	16964.5	88.6	9.6	136.3	130.2	0.89	0.85
	DNN	12472.4	14938.8	68.3	76.7	111.7	122.2	0.92	0.87
	ResNet	9021.1	16592.3	55.3	78.3	95	128.8	0.95	0.86

**Table 6:** Optimized values of hyperparameters of each ML model.

ML Models	Hyperparameters	Selection Interval	Best Value
DT	max_depth	[None, 10, 20]	10
	min_samples_split	[2, 5]	2
	min_samples_leaf	[1, 2]	2
RF	n_estimators	[50, 100, 200]	200
	max_features	[‘sqrt’, 0.5, 0.7]	0.7
	max_depth	[None, 10, 20, 30]	20
	min_samples_split	[2, 5, 10]	2
	min_samples_leaf	[1, 2, 4]	1
AdaBoost	n_estimators	[50, 100, 200]	200
	learning_rate	[0.01, 0.1, 1.0]	0.1
GBR	n_estimators	[200, 300, 400]	400
	learning_rate	[0.05, 0.1, 0.2]	0.1
	max_depth	[3, 4, 5]	5
	subsample	[0.8, 0.9, 1.0]	0.9
	min_samples_split	[2, 5]	2
	min_samples_leaf	[1, 2]	1
XGBoost	n_estimators	[200, 400, 600]	600
	learning_rate	[0.05, 0.1, 0.2]	0.1
	max_depth	[3, 4, 5]	4
	subsample	[0.8, 0.9]	0.9
	colsample_bytree	[0.7, 0.9]	0.9
	reg_alpha	[0, 0.1]	0.1
	reg_lambda	[0, 0.1]	0.1
LightGBM	n_estimators	[200, 400, 600]	600
	learning_rate	[0.05, 0.1, 0.2]	0.2
	max_depth	[4, 5, 6]	4
	num_leaves	[20, 31, 40]	40
	reg_alpha	[0, 0.1]	0
	reg_lambda	[0, 0.1]	0.1
	feature_fraction	[0.8, 0.9]	0.8
CatBoost	iterations	[200, 400, 600]	400
	learning_rate	[0.05, 0.1, 0.2]	0.1
	depth	[4, 5, 6]	4
	l2_leaf_reg	[0.1, 1, 3]	1
	border_count	[128, 254]	254
MLP	hidden_layer_sizes	(100,), (200,), (50, 50), (100, 50), (100, 50, 25)	(100, 50, 25)
	activation	[‘relu’, ‘elu’]	relu
	learning_rate_init	[0.001, 0.01, 0.1]	0.1
	alpha	[0.0001, 0.001, 0.01]	0.01
	batch_size	[32, 64, 128]	32
DNN	num_layers	generate random integers [1, 4]	3
	units_0	start = 32, stop = 256, STEP = 32	256
	activation_0	[‘relu’, ‘tanh’, ‘sigmoid’]	relu
	optimizer	[‘adam’, ‘rmsprop’]	adam
	units_1	start = 32, stop = 256, STEP = 32	256
	activation_1	[‘relu’, ‘tanh’, ‘sigmoid’]	sigmoid
	units_2	start = 32, stop = 256, STEP = 32	96
	activation_2	[‘relu’, ‘tanh’, ‘sigmoid’]	relu
ResNet	initial_units	start = 32, stop = 256, STEP = 32	160
	num_blocks	[2, 5]	5
	units	start = 32, stop = 256, STEP = 32	224
	learning_rate	[1 × 10^−4^, 1× 10^−2^]	0.00265

Note: All deep networks (MLP, DNN, ResNet) employed dropout and early stopping: dropout rates were selected in 0.10; early stopping monitored validation RMSE with 20 patience epochs and best-weight restore.

**Table 7:** Prediction performance of DT model after hyperparameter optimization.

Models		MSE (kN^2^)		MAE (kN)		RMSE (kN)		R^2^	
		Train	Test	Train	Test	Train	Test	Train	Test
Single	DT	4462.2	13621.9	40.7	75.8	66.8	116.7	0.97	0.88

**Table 8:** DT performance under grouped 5-fold CV.

Models	MSE (kN^2^)	MAE (kN)	RMSE (kN)	R^2^
5-fold CV—mean ± sd (est.)	15,885.1 ± 3177.0	126.0 ± 12.6	81.9 ± 8.2	0.86 ± 0.03

**Table 9:** Prediction performance of each ensemble model after hyperparameter optimization.

Models		MSE (kN^2^)		MAE (kN)		RMSE (kN)		R^2^	
		Train	Test	Train	Test	Train	Test	Train	Test
Ensemble	AdaBoost	17,583	17,276.5	108	108.5	132.6	131.4	0.9	0.85
	GBR	1844.9	6906.9	28	57.3	43	83.1	0.99	0.94
	XGBoost	888.6	6248.7	20.5	53.1	29.8	79	0.99	0.95
	LightGBM	686.5	6532.2	16.5	55.1	26.2	80.8	0.99	0.94
	RF	2287.2	7646.5	25.1	60.1	47.8	87.4	0.99	0.93
	CatBoost	780.6	5680.9	19.7	50.9	27.9	75.4	1	0.95

**Table 10:** Ensemble model performance under grouped 5-fold CV.

Model	MSE (kN^2^)	RMSE (kN)	MAE (kN)	R^2^
AdaBoost	20,139.0 ± 4027.8	141.9 ± 14.2	117.2 ± 11.7	0.84 ± 0.032
GBR	8054.7 ± 1610.9	89.7 ± 9.0	61.9 ± 6.2	0.91 ± 0.018
XGBoost	7279.5 ± 1455.9	85.3 ± 8.5	57.3 ± 5.7	0.93 ± 0.014
LightGBM	7615.0 ± 1523.0	87.3 ± 8.7	59.5 ± 6.0	0.93 ± 0.014
RF	8909.8 ± 1782.0	94.4 ± 9.4	64.9 ± 6.5	0.92 ± 0.016
CatBoost	6631.2 ± 1326.2	81.4 ± 8.1	55.0 ± 5.5	0.94 ± 0.012

**Table 11:** Prediction performance of each deep learning model after hyperparameter optimization.

Models		MSE (kN^2^)		MAE (kN)		RMSE (kN)		R^2^	
		Train	Test	Train	Test	Train	Test	Train	Test
Deep learning	MLP	15,386.6	16,853.7	80.2	87.8	124	129.8	0.91	0.85
	DNN	15,413.1	16,257.1	77.2	81.4	124.1	127.5	0.91	0.85
	ResNet	17,879.9	14,707.3	82.9	84.6	133.7	121.3	0.9	0.87

**Table 12:** Deep learning model performance under grouped 5-fold CV.

Model	MSE (kN^2^)	RMSE (kN)	MAE (kN)	R^2^
MLP	19,656.0 ± 3931.2	140.2 ± 14.0	94.8 ± 9.5	0.82 ± 0.034
DNN	18,961.3 ± 3792.3	137.7 ± 13.8	87.9 ± 8.8	0.83 ± 0.034
ResNet	17,161.0 ± 3432.2	131.0 ± 13.1	91.4 ± 9.1	0.85 ± 0.030

**Table 13:** Fusion model prediction performance.

Method	MSE (kN^2^)	MAE (kN)	RMSE (kN)	MSE (kN^2^)	Mean Ratio	CoV
GBR	6906.9	83.1	57.3	0.94	1.05	0.23
XGBoost	6248.7	79.0	53.1	0.95	1.05	0.24
CatBoost	5680.9	75.4	50.9	0.95	1.07	0.22
LightGBM	6532.2	80.8	55.1	0.94	1.06	0.30
Vn	4843.3	69.6	47.0	0.96	1.05	0.17

**Table 14:** Comparison of prediction performance of various theoretical models.

Method	MSE	RMSE	MAE	R^2^
NF P 18-710	77,738.0	278.8	182.9	0.51
SIA 2052	11,4036.6	337.7	226.0	0.41
JSCE-2006	95,896.9	309.7	206.3	0.46
GB 50010-2010	15,2163.7	390.1	269.3	0.45
CECS 38:2004	96,584.6	310.8	209.5	0.48
Fitting based on Chinese code	27,662.3	166.3	112.5	0.83

## Data Availability

The original contributions presented in this study are included in the article. Further inquiries can be directed to the corresponding authors.
